# Mastoid Obliteration with the “Cupeta Technique” After Canal Wall Down Tympanoplasty in Chronic Otitis Media with Cholesteatoma: Preliminary Results

**DOI:** 10.3390/biomedicines13102391

**Published:** 2025-09-29

**Authors:** Antonio Faita, Gian Marco Volpato, Diletta Trojan, Giulia Montagner, Valerio Maria Di Pasquale Fiasca

**Affiliations:** 1 Section of Otorhinolaryngology, Cittadella Hospital, 35013 Cittadella, Italy; 2 Section of Otorhinolaryngology, Department of Neurosciences, University of Padova, 35121 Padua, Italy; gianmarco.volpato@aopd.veneto.it (G.M.V.); valeriomaria.dipasqualefiasca@aopd.veneto.it (V.M.D.P.F.); 3 Fondazione Banca Dei Tessuti Del Veneto, 31100 Treviso, Italy; dtrojan@fbtv.it (D.T.); gmontagner@fbtv.it (G.M.)

**Keywords:** mastoid obliteration, chronic otitis media, canal wall down tympanoplasty, homologous bone, Palva flap

## Abstract

**Background/Objectives**: Mastoid obliteration (MO) after canal wall down (CWD) tympanoplasty for chronic otitis media with cholesteatoma (COMC) enables simultaneous surgical management of the pathology and shaping of a new external ear canal (EEC) that is similar to the natural one. The aim of the present work is to describe the results of a new MO technique that involves using homologous bone (HB) material and a Palva flap (“Cupeta technique”). **Methods**: A retrospective study was conducted on 12 patients undergoing MO for COMC, either during the same operation or in a second-time surgery after CWD. The surgical technique, patient demographics, audiometric data, the EEC volume, and clinical outcomes were analyzed. **Results**: The MO technique resulted in good outcomes in terms of healing at three months after surgery. Fewer clinical complications were observed compared with similar MO methods described in the literature. HB reabsorption was observed in two patients and was defined as only partial. Measurements of the EEC volume were normal in all patients. The preoperative and postoperative hearing thresholds were similar. **Conclusions**: Performing MO with the Cupeta technique after CWD is a suitable surgical management method for COMC and demonstrates good clinical postoperative results. We plan to conduct further studies with a longer follow-up and a larger group of patients in order to confirm our findings.

## 1. Introduction

The concept of mastoid obliteration (MO) was introduced for the first time in 1911 and has recently become popular. Subsequently, Palva introduced the use of autologous bone for MO in combination with a muscle-periosteal flap. In the last two decades, the safety of the MO procedure has increased. One of the reasons for this is the introduction of non-EPI DWI (echo-planar imaging–diffusion weighted imaging) magnetic resonance imaging (MRI) sequences, which have enabled the non-surgical follow-up of patients treated for chronic otitis media with cholesteatoma (COMC), thus avoiding problematic second looks at obliterated cavities.

COMC is the most aggressive type of chronic otitis. It is characterized by the presence of a keratinizing squamous epithelium within the middle ear and mastoid. The cholesteatoma matrix can produce an inflammatory reaction that is capable of eroding the bony structures of the temporal bone, causing partial or total deafness, facial nerve damage, and local and intracranial infections. Treatment of COMC is surgical and must be as radical as possible. The main goal of this type of surgery is to achieve a disease-free and dry ear, and where possible, restore functional hearing and achieve adequate esthetic results [1].

There are multiple surgical approaches for COMC, and when choosing which one to use, several factors should be considered. The main characteristics influencing the choice of an appropriate surgical approach are the position of the cholesteatoma, the extension, and the residual hearing function. The conservative approach is canal wall up (CWU) tympanoplasty, which entails the preservation of the EEC and the volume of the middle ear. With this approach, the ossicular reconstruction is generally more stable than that achieved with other, less conservative techniques, thanks to the preservation of the original bony annulus, which helps to stabilize the prosthesis.

Canal wall down tympanoplasty (CWD) involves drilling of the posterior and superior walls of the EEC. This procedure allows for the shaping of a common cavity between the EEC and the mastoid, a newly small middle-ear cavity. Adequate reshaping of the concha and meatoplasty must be performed in order to achieve an efficient self-cleaning cavity. Despite being more aggressive toward the ear structures, CWD provides relevant surgical benefits. It allows for total visualization of the middle-ear pathology in the deep tympanic sinus or anterior epitympanum. Historically, CWD is associated with clinical postoperative disadvantages such as the need for frequent follow-up evaluation to clean the mastoidal cavity, ear water intolerance, blemishes of the EEC, recurrent infections, and potential difficulties in fitting hearing aids. Nonetheless, if properly performed, outcomes in terms of quality of life do not seem to differ greatly between CWD and CWU.

Performing the MO procedure after CWD allows for the shaping of a new EEC that closely resembles the preoperative EEC, yielding significant advantages [2]. MO can be performed in a single stage along with CWD, or in a second stage. The second-stage approach may be taken for multiple reasons: suspected recurrence of cholesteatoma, chronic otorrhea, persistent episodes of vertigo, unstable ears with cleaning difficulties, and the need for a follow-up surgical procedure for COMC.

Several surgical options and materials are available for performing MO, with no consensus on the standardization of procedures. Furthermore, obliteration techniques include the use of various local flaps, with or without combinations of “free-in-the-cavity” materials. Materials used for obliteration can be divided into organic and synthetic materials. Organic material can be divided into autologous (e.g., bone, cartilage, muscle, fat) and heterologous (bio-hydroxyapatite from animal origin) materials. Various synthetic materials may be used including hydroxyapatite, calcium phosphate ceramic, bioactive glass, silicone, and titanium [3,4].

Homologous bone (HB) is tissue collected from cadaver donors and is a relatively new entry in our field. It has already been used for reconstructive procedures in other anatomical regions. The safety and efficacy of HB have been reported in dentistry, maxillofacial surgery, and orthopedics. A bone tissue graft is harvested from cadaver donors and suitably reduced into freeze-dried granules. Using HB instead of autologous bone avoids surgical damage to the donor site, and it has osteoconductive properties and lacks immunological response or signs of inflammation [5,6]. 

In the present study, we introduce our series of patients treated with MO, in association with or secondary to CWD, with or without simultaneous ossicular reconstruction. The MO technique was carried out using HB, assisted by positioning a Palva flap. We named this MO procedure the “Cupeta technique”.

## 2. Materials and Methods

We present a retrospective review of 12 patients who were treated for COMC with MO using HB and a Palva flap in the Ear, Nose, Throat (ENT) unit of the Cittadella Hospital between 2019 and 2023. The study was approved by the Institutional Ethics Committee of Central Eastern Veneto Region on 7 November 2024 (approval number 0190251/2024). MO was performed simultaneously with CWD (in five cases) or in a second stage (in seven cases) after CWD. Simultaneous MO and CWD was indicated to manage the presence of cholesteatoma. Second-stage MO was performed as a second-look stage in unstable ears or in the case of a clinical or radiological suspicion of cholesteatoma recurrence. 

### 2.1. Surgical Technique

The surgical procedure of MO follows the same steps reported in a previous study by our group [7]. Before the procedure, a skin plasty of the external third of the EEC (meatoplasty) is performed. Subsequently, two triangular local flaps are shaped, one inferiorly hinged and the other anteriorly hinged, without sacrifice of the cartilage of the concha. The flaps are sutured medially and posteriorly with the muscle-periosteal Palva flap, elevated previously while managing the retroauricular tissues in the surgical access. The Palva flap is classically anteriorly based; therefore, during the meatoplasty, the flap’s pedicle must be carefully preserved. Our HB was a freeze-dried corticocancellous particulate of human bone. On average, 3 cc fine granules (dimensions of 125–850 μm) were used in most cases. HB was mixed with 2 mL of human fibrin glue (Tissucol, Tisseel, Baxter, Glenview, IL, USA) in a sterile bowl to obtain a homogeneous and malleable compound. Subsequently, it was divided into rectangular or squared blocks (with dimensions varying from 2 to 7 mm). The blocks, which were initially malleable but then hardened due to a change in the consistency of the fibrin glue, were introduced separately into the cavity, obliterating the whole mastoid from medially to laterally including the epitympanum, superior to the second tract of the facial nerve. The obliteration was implemented anteriorly until a normal-sized EEC was newly shaped. The anterior level of obliteration corresponds to the new posterior wall of the EEC. We named the preparation and the use of the material in this procedure the “Cupeta technique”. The Palva flap was flipped anteriorly to the obliteration, aiming to promote the new EEC’s re-epithelialization and protect the bony obliteration from external factors or possible reabsorption. The Palva must be sufficiently long to cover the new EEC’s medial area up to the epitympanum area exposed during the CWD. If the Palva has insufficient length, other ancillary flaps can be used medially (temporalis fascia free flap, bovine pericardium patch, etc.). The healing of neo-EEC was supported for the following 15 days with a sterile gelatine sponge medication (Spongostan™, Ethicon, Raritan, NJ, USA) in the medial part, comprehensive of the neo-tympanum, and with an iodoform gauze impregnated laterally with antibiotic ointment.

### 2.2. Tissue Supply and Processing

The “Fondazione Banca dei Tessuti del Veneto”, a non-profit institution accredited by the National Transplant Center and Regional Competent Authority, provided the human bone tissue used in the study. Homologous bone tissues were retrieved from cadaver donors before serological and molecular testing according to Italian requirements, after the acquisition of proper informed consent; the entire process was conducted in the tissue bank’s Good Manufacturing Practice (GMP)-compliant facility. The process involved the following steps: the distal portion of the femur and the proximal portion of the tibia, from which the freeze-dried corticocancellous bone particles were obtained, then retrieved in an operating theater and immersed in antibiotic solution for a first round of decontamination [8,9]. Subsequently, the bones were transported at refrigerated temperature to the tissue bank facility, where they were aseptically processed to remove eventual soft tissue residues and then decontaminated again. The proper bone tissue was ground with a Fortios Bone Mill (Spierings Orthopaedic, Nijmegen, The Netherlands), and afterward, blood and lipid residues were melted with ethanol and hydrogen peroxide (Carlo Erba, Cornaredo, Italy), through a protocol adapted from Eagle et al. [10] The resulting morcellized bone was then dried for eighteen hours in a freeze dryer (Scientific Products, Warminster, PA, USA). Constantly, through the entire process, several microbiological tests were conducted to verify compliance with established regulatory criteria. In accordance with the manufacturer’s instructions (BD, Becton, Dickinson Company, Franklin Lakes, NJ, USA), samples were inoculated and incubated in specific BD BADTEC culture vials. It was determined that the residual moisture content should be within the recommended range of 1–6% (“Guide to the quality and safety of tissue and cells for human application”, European Directorate for the Quality of Medicines and Healthcare), and this was checked in each freeze-dried bone batch before tissue release for transplantation. Finally, the product was packaged and stored between +15° and +25° Celsius until use. The entire process was approved by the Italian competent authority that authorized the distribution of HB.

### 2.3. Recorded Data

The following preoperative data were retrieved: demographic data (gender, age), general and ontological anamnesis, preoperative hearing thresholds with pure-tone audiometry, and endoscopic evaluations. Other surgical information collected included the type of surgery (CWD + MO, MO in second look), the position of the cholesteatoma in the subzones (mastoid, epitympanum, mesotympanum, hypotympanum, external canal), and ossicular reconstruction. After discharge from the hospital, patients underwent sequenced postoperative follow-up. Short-term complications at 1 month and 3 months as well as postoperative short-term pure-tone hearing thresholds were reported. Statistical analysis was performed with the Wilcoxon signed-rank test and paired *t*-tests. Impedancemetry was performed, and the postoperative EEC volume was calculated. The average EEC volume in adults ranges from 0.6 cm^3^ to 2.5 cm^3^; values superior to 2.5 cm^3^ are considered suggestive of eardrum perforation.

## 3. Results

In the evaluated sample, seven patients had secondary surgery after a first-step surgery (six CWD, one CWU) indicated for COMC. Five patients had primary surgery with a diagnosis of COMC (Table 1).

The seven secondary surgeries were motivated by clinical symptoms, mainly chronic infection (7/7) and otorrhea (5/7); no postoperative facial palsy was observed. Among these revision patients, three had recurrent cholesteatoma, while four of them (patients n° 3, 4, 5, and 10) showed no residual disease and underwent MO alone; three of them also received ossiculoplasty.

Preoperative and postoperative symptoms observed during the follow-up assessments at 1 and 3 months are described in Table 2. All patients presented at least one symptom among pain, chronic ear infection, persistent visible otorrhea, tinnitus, and vertigo before the operation. Tinnitus was present in only one patient. Regarding EEC, the median postoperative EEC volume was 2.13 cm^3^ (range 1.46–2.33 cm^3^), which is below the normal threshold of 2.50 cm^3^. Partial extrusion was observed in patient no. 7 at one month and persisted at three months, while in patient no. 10, the phenomenon appeared at the three-month follow-up. In the former patient, this complication caused delayed healing with otorrhea, pain, and persistent inflammation during the first month, but complete resolution was achieved by three months; the latter showed persistent infection after three months, requiring prolonged topical antibiotic therapy, although his final EEC volume was comparable to that of the rest of the cohort.

Data regarding the surgical technique and cholesteatoma position are reported in Table 3.

All patients underwent obliteration; eight received tympanoplasty (primary or secondary) and MO, three received MO and ossiculoplasty, and one underwent MO alone.

Ossiculoplasty was performed when reconstruction of the ossicular chain was feasible and/or necessary. Seven patients received the procedure: three (no 2-6-11) in the first surgery, and four (no 4-5-10-12) in the second look. Patient nos. 1–3 had already received ossiculoplasty and required no further reconstruction, while patient nos. 9–10 had only partial ossicular sacrifice without the need of a new interposition in the ossicular chain. In patient no. 7, ossiculoplasty was not feasible due to a facial nerve dehiscence hindering the oval window view.

The most frequently applied type of prosthesis was a titanium total ossicular reconstruction prosthesis (TORP), which was positioned in five patients (41.7%), while a partial ossicular reconstruction prosthesis (PORP) was used in one case, and columellar cartilage upon a mobile stape was also used in one case (TORP and PORP, Audio Technologies, Gossolengo, PC Italy).

Hearing thresholds were measured before surgery, at 1 month and 3 months postoperatively, and these data are reported in Table 4. Statistical analysis with the Wilcoxon signed-rank test and paired t-tests revealed no significant differences between the preoperative and postoperative values, except for BC PTA at three months postoperatively (*p* = 0.038).

## 4. Discussion

The question of which is the best practice for cholesteatoma treatment among the CWD and CWU techniques is a source of debate among otosurgeons. The choice to use CWD in primary surgery in our series was motivated by the aim of achieving the optimal management of COMC. A critical area of focus for managing cholesteatoma is the epitympanum; this subzone was defined in all five patients who underwent MO during primary surgery. Location in the mastoid was present in all cases; patient nos. 6–8 also had location in the mesotympanum. Regarding the second-step surgery, indication of this approach was motivated by clinical symptoms, chronic infection, and otorrhea. Three patients were revealed to have cholesteatoma. These data highlight that symptoms alone are not a reliable predictor of the recurrence of COMC, but they must be carefully considered if they are present for a long time. At our center, the use of a two-stage approach for cholesteatoma is uncommon, and follow-up is usually based on non-EPI DWI MRI at 3 and 5 years, and not before this, since recurrent cholesteatomas must be large enough to be seen on MRI. We believe that MRI performed in the first two years may lead to false positives for cholesteatoma, as is also reported in the literature [11].

Patient no. 12 had a conversion from primary CWU to CWD because of erosion of the EEC caused by the cholesteatoma. The use of both CWD and MO could represent, in our opinion, an approach that combines the advantages of the two types of tympanoplasty. CWD ensures wide exposure of the middle-ear structures and mastoid; MO restores the anatomic boundaries, aiming to achieve a neo-EEC and restore near-normal external ear anatomy and “mimics” the results of CWU. The use of CWD with MO diminishes the risk of persistent otorrhea and infections as well as reducing the buildup of cerumen within the neo cavity and the need for frequent follow-up evaluations for ear cavity cleaning; it also reduces the susceptibility of the ear to vertigo induced by caloric stimulus. Furthermore, it has a beneficial influence on the recurrence rate of cholesteatoma compared with the outcomes of CWU mastoidectomy [12,13,14] and CWD without MO (recurrence in CWD 16.5% vs. CWD+MO 5.3%) [15]. MO has clinical postoperative outcomes that have been reported to play a role in reducing the symptoms experienced by patients after otologic surgery.

MO can be performed in various surgical scenarios in addition to CWD: mastoidectomy, cochlear implantation, or subtotal petrosectomy. In each of these surgeries, it may be applied simultaneously with these procedures or as a second-stage surgery.

Surgical strategies for MO are various and can be combined, as in our series. We used “free-in-the-cavity” HB combined with fibrine glue, together with a Palva flap, sometimes reinforced with fibrous patches and/or free temporal muscle fascia.

Pedicled local flaps have a historical and current role in MO: these flaps can function as a conductive surface, promoting the re-epithelization of the new EEC. Several types of flaps have been described so far. A combination of flaps and MO techniques can be employed in order to achieve optimal obliteration of the cavity. The Palva flap was introduced in the XX century (it has been used since the 1950s) and is probably the most well-known flap used in otological surgery. It is an anteriorly hinged—close to the external acoustic meatus—and muscle-periosteal flap that is positioned in the cavity for its obliteration. As suggested by the author himself, bone pate/chips can be previously positioned in the cavity, further diminishing the effects of subsequent atrophy of the muscular component. We agree that this is a suitable surgical approach. Palva flaps facilitate healing and prompt re-epithelization of the EEC, thereby averting exposure of the bone allograft to the external environment. Moffat introduced a variant of the Palva flap with a superior-based hinge [16]. This postauricular periosteal–pericranial flap, unlike Palva’s, consists only of the periosteum and pericranium, and is obtained through elevation of the temporalis muscle. It is an inferiorly based flap that can be very long, and can also be combined with bone pate. The temporo-parietal fascial flap is similar to the PPPF, but presents a vascular pedicle, based on the superficial temporal artery; it is more resistant to infection and useful in conditions such as devascularized bed tissues (e.g., after radiotherapy) and in revision surgeries. The Hong Kong flap, similar in concept to the Palva flap but with a wide anterior hinge (not limited to the meatus), allows further material to be used for the obliteration, if needed. It requires an endaural superior extension and intertragal incision, making it compatible with some surgical openings. A slightly different option is the postauricular myo-cutaneous flap, which uses postauricular skin, the periosteal sternocleidomastoid muscle, and a soft tissue paddle based on the auricular branch of the occipital artery. If the PMC flap is used, incision lines must be preoperative planned.

Other flaps include the middle temporal artery flap, the temporalis muscle flap (more easily used in oncological surgery), and the inferiorly based fascio-periosteal flap.

Free-in-the-cavity materials are divided into organic (autologous and homologous bone) and biomaterial–synthetic materials. The “perfect” material, in our experience, should be biocompatible, with no tendency for extrusion or reabsorption; cheap; easy to remove in revision surgery; detectable on CT or MRI; sufficient in quantity; and have osteoconductive properties.

Comparing results published in the literature for the use of materials is a difficult task for the following reasons: (1) the paucity of publications for each material and the small number of cases per series; (2) a lack of description of the mastoid volume to be obliterated, which is a key finding for understanding the shortness of autologous material; (3) a lack of studies demonstrating, in vivo and on cadavers, the osseointegration parameter in the mastoid (unlike other anatomical regions of maxillofacial competence); (4) no possibility of economic analysis, given the extreme variability in the cost of each individual material across countries because the operating room costs are not calculated for the time spent on the procedure and the cost of ancillary materials (such as Tissucol in our article), and because the authors never describe the quantity of material required for each surgical procedure. Regarding the latter issue, when comparing current prices on the Italian market of resources for hospitals in the Italian health system, we can only say that for our Cupeta technique, 3 cc of HB is needed, which is similar to the quantity of hydroxyapatite required, but HB is cheaper, even when considering the cost of the human fibrin glue used to create the compound used in this technique, whilst bioactive glass is more expensive, regardless of the quantity used. Nevertheless, there is not yet a standardized procedure for choosing the right material, as no statistically significant difference in output has been detected. Therefore, we provide a brief description of each material and a summary of truly comparable data in Table 5.

### 4.1. Biomaterials

Synthetic materials typically possess biocompatibility and facilitate re-ossification. Nevertheless, potential drawbacks associated with such materials include middle-ear or bone infections and graft extrusion during medium- or long-term follow-up [23].

- Bioactive Glass

Bioactive glass (S53P4-Bone Alive^®^ Turku, Finland) has garnered attention in recent studies. Composed of glass granules, it can chemically bond with the surrounding bone, thereby fostering new bone formation in the reconstruction site. A study conducted by Fassone et al. estimated that this glass has a re-epithelization period of 45 days, coupled with low complication rates [24]. Its extrusion ranges from 0.5% to 3.3% [17]. Furthermore, the contamination of bioactive glass can be reduced with keratin-producing cells, the glass can be easily removed for revision surgery and has antibacterial properties. In a relevant study on the material, a 5-year recidivism rate of cholesteatoma of 12% was observed, and a Marchant grade of 0 to 1 was achieved by 95% of patients [16]. Mishra et al. reported that bioactive glass had the same efficacy as autologous bone [25].

- Silicone and Titanium Mesh

Silicone and titanium are pure synthetic materials that are rarely applied. There is only one small series for titanium mesh [18]. Silicone has been employed in the form of small blocks for reconstruction following CWD mastoidectomy due to its cost-effectiveness; however, it may incite a foreign-body reaction in predisposed patients [5]. Its extrusion rate is similar to that of bioactive glass [4].

- Heterologous Bone

Hydroxyapatite (heterologous bone) is harvested from cortical bovine bone, maintaining only the bony structure without active cells, so it can be considered synthetic and biological. It has been extensively utilized for MO, being renowned for its superior osteoconductivity among non-autologous materials. In a recent study by Sahli-Vivicorsi comparing radiological differences between bioactive glass and hydroxyapatite, the two materials showed feasibility and long-term stability, with hydroxyapatite demonstrating better osteointegration and comparable reabsorption [26]. This is the only study we are aware of on osteointegration in the mastoid. Compared with the use of autologous bone, MO with hydroxyapatite seems to be safer and more effective, with higher success rates in achieving a dry ear, lower recidivism rates, and better hearing outcomes at short-term follow-up [19]. Hydroxyapatite could have a similar extrusion range to other biomaterials, but in Skoulakis’s review, the most exhaustive review on this topic, the results were conditioned by the Ridenour study (100% extrusion) [17,20].

### 4.2. Organic Bone

- Autologous Bone

Autologous bone pate was for a long time the only organic bone material available, and continues to be the reference material in otosurgery. It is typically obtained from cortical drilling during mastoidectomy and can be easily packed into the cavity to restore its contour. Several studies report that this material has high success rates and very low graft failure compared with synthetic substitutes [23]. Compared with homologous bone or biomaterials, autologous bone shows a theoretical lack of extrusion but a higher tendency for resorption when postoperative infections occur. Resorption can occur anyway over time, particularly in large or revision cavities, potentially compromising long-term stability [23,27]. The most frequent clinical complication is persistent otorrhea, described in up to 10–20% of cases, which sometimes requires prolonged topical therapy or revision surgery [16,23,27]. The principal limitation in clinical practice is the restricted availability of material, especially in pediatric or revision cases [17]. Despite these issues, bone pate remains widely used in primary mastoid surgery, where its integration capacity and low cost make it a reliable first-line option [16,23,27].

- Homologous Bone

In other anatomical districts, compared with non-bone biomaterials, HB shows naturally superior osteointegration, osteoconductivity, and the absence of immunological reactions or inflammation [5,6]. Moreover, it has been reported that processes that remove or destroy the cells of the donor tissue, such as washing steps with water or solvent and freeze-drying, tend to reduce the antigenicity associated with the processed graft [28]. The washing protocol of HB was adapted from the procedure described by Eagle et al., which is efficient in the removal of DNA [10].

The use of HB in MO is reported only in our previous case report [7], in which the material did not demonstrate reabsorption and no clinical complications occurred, and in two recent studies by Fieux et al. and Kim et al. [21,22]. The comparison of HB with other materials is limited by the small number of studies involving homologous bone, but the results in terms of clinical complication and extrusion rates seem quite similar to those for other materials.

We now compare the two published studies involving HB with our case series with regard to the features of the material, surgical procedure, clinical outcomes/complications, reabsorption, and hearing outcomes.

- Features of HB

Fieux used cancellous bone dust, referred to as allograft bone dust (AG) Phoenix^®^ (5–7 cc bone graft chips, TBF, Moins, France). The average amount of material used was not reported, nor the granular size of the material, nor whether it was ground into dust by a surgical instrument [21].

Kim used coarse cancellous bone crushed to 3 mm in diameter (coarse cancellous; Maxxeus^®^, Kettering, OH, USA); no other information was reported. We used, on average, 3 cc fine granules of freeze-dried corticocancellous bone (125-850 μm dimension of granules). HB was mixed with 2 ml of human fibrin glue (Tissucol^®^ Baxter, Deeerfield, IL, USA) and blocks 2–7 mm larger in size. These blocks were even smaller in size and physically smoother than the granules used in other studies, thanks to the use of both cortical and cancellous bone, and especially thanks to mixing the bone with fibrin glue [22].

- Surgical Procedure

In the study by Fieux, MO was performed in CWD with HB material for pure MO, and the posterior wall of the EEC was reconstructed with autologous concha cartilage. Ossiculoplasty is cited, but no details are reported [21]. Kim used HB for MO and for the shaping of the EEC, along with free temporalis fascia to protect the material from the cavity of the neo-EEC. There is no mention of ossiculoplasty [22]. In our study, we used blocks of HB for MO and shaping of the EEC; coverage of the EEC was guaranteed by the temporalis fascia or Palva flap. Our technique is similar to Kim’s technique, but we believe that administering HB in blocks, without free granules, can ensure greater control over the placement of the material in the mastoid cavity and avoid affecting the facial nerve canal, which must be left untouched by bone remodeling to ensure greater safety in any revision surgery. Furthermore, in our opinion, the use of blocks rather than dust or granules may pose a lower risk of extrusion in the short-term. This is the peculiarity and uniqueness of the “Cupeta technique”. Ossiculoplasty is performed when necessary (not in some secondary surgeries) and when anatomically possible to do, with the use of prostheses (TORP or PORP) or autologous material (cartilage).

- Clinical outcomes and reabsorption

Fieux compared two groups of patients with a diagnosis of COMC who received MO surgery with bioactive glass vs. HB after CWD tympanoplasty (17 with HB, and a total of 32 patients). There is no indication of whether this was primary or secondary surgery. The clinical follow-up was 18 months. The number of patients with MO-HB with complications was 40% (four with otorrhea, one with material extrusion, and two with residual cholesteatomas) [21].

Kim’s study presented 23 patients with COMC or otitis without cholesteatoma undergoing CWD as primary or secondary surgery. The follow-up was 38.9 months. Clinical complications occurred in four cases (23%) with one case of otorrhea, two cases of retraction pockets, and one case of tympanic perforation [22].

Our group of patients (12) presented with the same pathology (COMC). Follow-up was limited (3 months). Two patients had complications at 3 months (16.7%), with both presenting only partial reabsorption of obliteration. Patient no. 7 had reabsorption due to infection that was not evident at 3 months, and patient no. 10 had reabsorption with infection present at 3 months, but without otorrhea. We should add, for the record, that patient no. 10 no longer had infection at 6 months. For the patient who had tinnitus at 3 months, this cannot be considered a real complication, since the tinnitus, which was already present preoperatively, was not definitively attributable to COMC. We consider only “partial” reabsorption to have occurred because the EEC volume of all patients was within the normal limits, below the theoretical maximum limit of normality of 2.50 cm^3^. We consider the EEC volume, which has never been presented in the literature before, to be a reliable parameter of reabsorption and more important in clinical practice than osseointegration or osteoconductivity, since it is the EEC volume that ensures normal healing and clinical well-being for the patient.

- Hearing Outcomes

In the series of Fieux, there were no significant differences between the preoperative and postoperative hearing outcomes [21]. Kim obtained hearing results without statistical significance, but only a tendency toward a reduction in the air bone gap [22].

In our study, we also found no statistically significant differences in the hearing threshold. We can descriptively indicate an improvement in the first postoperative month, and a confirmation, after three months, of the same AC and BC PTA. We did not stratify the hearing results according to the ossiculoplasty performed due to the small number of patients in the series and the fact that there were two biases: some patients did not undergo ossiculoplasty because it had been performed in the previous surgery, and one patient had an anatomical contraindication to undergoing it.

We also affirm that ossiculoplasty could also be considered carefully in patients with recurrent ear infections or chronic otorrhea, because in these cases, the surgeon cannot be completely sure that the middle ear has been cleaned of cholesteatoma.

Many recent studies have investigated the hearing threshold following MO [1,29] and found no evidence of any difference between CWD and CWD with MO. We believe that hearing threshold results should not be a determining factor in choosing whether to perform MO, particularly in CWD, since the condition of the ossicular chain and the transmission of sound between the eardrum and the oval window are minimally affected by obliteration. Otherwise, restoring a normal EEC would improve hearing quality, as has been demonstrated by studies on EEC resonance [30] and real ear unaided response (REUR) [31]. A recent study by our group demonstrated good hearing wellness in subjective analysis (answers to the speech, spatial and quality aspects of the hearing scale questionnaire—SSQ) [32].

## 5. Conclusions

CWD with MO is a technique that achieves good surgical control of pathology, especially in COMC and in critical areas of the middle ear, like the epitympanum, as highlighted in our series and in the literature. The cholesteatoma follow-up duration is up to 5 years, and our series should be observed for this length of time to be sure of no recurrence. The Cupeta technique is an MO approach in which the management of HB, together with covering the material with a Palva flap and free temporalis fascia when necessary, ensures the restoration of a normal EEC volume, with the advantages of a self-cleaning ear and imitation of a normal EEC. Free-in-the-cavity materials are difficult to compare, because studies do not analytically report the quantities of material used, there are no studies on osseointegration, and patient series typically include small groups. The cost-effectiveness of one material compared with another cannot be calculated, although we know that in the Italian economic market, HB is cheaper than other materials such as hydroxyapatite or bioactive glass. Despite these limitations, we observed that in the literature, HB demonstrates quite similar percentages to other materials in terms of clinical complications and extrusion. Although our clinical results can be considered early due to the short follow-up and small patient population, they are encouraging when compared to those of other published studies involving the use of HB, as clinical complications are fewer. The normal EEC volume observed indicated that reabsorption of the material was only partial in our series. Long-term observation of clinical and EEC results (≥2 years) is required. The Cupeta technique can be considered a novelty for its use of HB compared with other surgical techniques described in other studies. The EEC volume is an excellent parameter for evaluating the results of this approach, and we hope that it can be used in future studies for the evaluation of MO. The hearing results for MO and our technique, in particular, may be promising not in terms of the hearing threshold, but rather in terms of hearing quality.

## Figures and Tables

**Table 1:** General data and preoperative anamnesis.

#	Age (yrs)	Sex	Side	Previous	Type of Previous Surgery
1	11	M	L	Yes	CWD
2	51	F	L	No	Other
3	57	F	R	Yes	CWD
4	69	F	L	Yes	CWD
5	33	F	R	Yes	CWD
6	41	M	R	No	Other
7	74	F	R	Yes	CWD
8	40	M	R	No	Other
9	40	M	R	No	Other
10	66	M	L	Yes	CWD
11	21	M	L	No	Other
12	36	M	L	Yes	CWU
	Median 40.5 IQR 24	Male 58.3% Female 41.7%	Right 6 (50%) Left 6 (50%)	Total yes 7 (58.7%)	CWD 6 (50%) CWU 1 (8.3%)

CWD: canal wall down; CWU: canal wall up; IQR: interquartile range.

**Table 2:** Preoperative and postoperative clinical evaluation.

	Preoperative N (%)	Postoperative N (%)	
		1 Month	3 Months
Tinnitus	1 (8.33%)	1 (8.33%)	1 (8.33%)
Vertigo	1 (8.33%)	0	0
Chronic pain	1 (8.33%)	1 (8.33%)	0
Chronic infection	9 (75%)	1 (8.33%)	1 (8.33%)
Persistent otorrhea	6 (50%)	1 (8.33%)	0
Facial palsy	0	0	0
Heat sensibility	0	0	0
Obliteration reabsorption			2 (18.2%)
Mean external canal volume EEC (cm^3^)			1.96 (±0.47)
Median external canal volume EEC (IQR)			2.13 (1.46–2.33)

No. of patients = 12. IQR: interquartile range.

**Table 3:** Surgical technique and position of the cholesteatoma.

#	OPL	MO Without Tympanoplasty Surgery	Cholesteatoma Position				
			Mastoid	Epitympanum	Mesotympanum	Hypotimpanum	External Canal
1	No	No	Yes	Yes	No	No	No
2	TORP	No	No	Yes	Yes	No	No
3	No	Yes	No	No	No	No	No
4	PORP	Yes	No	No	No	No	No
5	TORP	Yes	No	No	No	No	No
6	Cartilage	No	Yes	Yes	Yes	No	No
7	No	No	Yes	No	No	No	No
8	No	No	Yes	Yes	Yes	No	No
9	No	No	Yes	Yes	No	No	No
10	TORP	Yes	No	No	No	No	No
11	TORP	No	Yes	Yes	No	No	No
12	TORP	No	Yes	No	No	No	Yes
	TORP: 6 (50%) * PORP: 1 (8.3%) *	4 (33.3%) *	7 (58.3%) *	6 (50%) *	3 (25%) *	0 (0%) *	1 (8.3%) *

* N(%). OPL: ossiculoplasty; TORP: total ossicular reconstruction prosthesis; PORP: partial ossicular reconstruction prosthesis.

**Table 4:** Preoperative and postoperative hearing evaluated with pure tone average (PTA).

#	Preoperative Hearing		Postoperative Hearing			
	AC PTA (dB)	BC PTA (dB)	1 mo. AC PTA (dB)	1 mo. BC PTA (dB)	3 mo. AC PTA (dB)	3 mo. BC PTA (dB)
Median (IQR)	43.12 (32.19–70.94)	19.375 (10.94–34.38)	33.75 (30.63–53.75)	18.75 (13.13–29.13)	44.37 (26.25–68.13)	20.62 (12.50–41.88)
Mean (SD)	51.15 (26.63)	23.73 (17.97)	42.84 (18.08)	22.45 (14.47)	48.98 (27.28)	28.18 (20.24)

No. of patients = 12. AC: air conduction; BC: bone conduction; PTA: pure tone average; IQR: interquartile range; SD standard deviation.

**Table 5:** Comparative analysis of free-in-the-cavity material.

Material	Extrusion Rate	Principal Clinical Complications
Bioactive glass (S53P4/45S5)	0.5–3.3% [17]	Othorrea 4–15% [17]
Silicone	3.5–5% [4]	Othorrea 1% [4]
Titanium mesh	0% [18]	0% [18]
Hydroxyapatite (heterologous bone)	5–15.8% [17] 0–20% [19]	Othorrea 19% [19] Revision surgery 100% [20]
Bone pate (autologous bone)	-	Othorrea 13.2% [19]
Homolgous bone	1.5% [21] 0% [22]	Othorrea 26.7% [21] Retraction pocket 8.7% [22]

## Data Availability

The raw data supporting the conclusions of this article will be made available by the authors on request.

## Abbreviations

The following abbreviations are used in this manuscript:

AC	Air conduction
BC	Bone conduction
COMC	Chronic otitis media with cholesteatoma
CWD	Canal wall down
DWI	Diffusion weighted imaging
EEC	External ear canal
ENT	Ear, nose, throat
EPI	Echo-planar imaging
HB	Homologous bone
IQR	Interquartile range
MO	Mastoid obliteration
MRI	Magnetic resonance imaging
PORP	Partial ossicular reconstruction prosthesis
PTA	Pure tone average
SD	Standard deviation
TORP	Total ossicular reconstruction prosthesis
